# Low Cardiorespiratory Fitness, Muscular Fitness, and Flexibility Are Associated with Body Fat Distribution and Obesity Risk Using Bioelectrical Impedance in Taiwanese Adults

**DOI:** 10.3390/ijerph19148858

**Published:** 2022-07-21

**Authors:** Hsiu-Hsi Chiang, Po-Fu Lee, Yun-Tsung Chen, Chi-Fang Lin, Shu Xu, Yi-Tien Lin, Yu-Ting Lin, Yan-Jhu Su, Ben-Chang Shia, Wen-Sheng ChangChien, Chien-Chang Ho

**Affiliations:** 1 Graduate Institute of Business Administration, Fu Jen Catholic University, New Taipei City 242, Taiwan; shc8091@gmail.com (H.-H.C.); 025674@mail.fju.edu.tw (B.-C.S.); 2 Department of Leisure Industry and Health Promotion, National Ilan University, Yilan County 260, Taiwan; pflee@niu.edu.tw; 3 College of Humanities and Management, National Ilan University, Yilan County 260, Taiwan; 4 Department of Physical Education and Sport Sciences, National Taiwan Normal University, Taipei City 106, Taiwan; cthero178@hotmail.com (Y.-T.C.); aa830521@gmail.com (C.-F.L.); 5 Department of Gerontology, University of Massachusetts Boston, Boston, MA 02125, USA; shu.xu@umb.edu (S.X.); yanjhu.su001@umb.edu (Y.-J.S.); 6 Superintendent Office, Fu Jen Catholic Hospital, New Taipei City 243, Taiwan; d00014@mail.fjuh.fju.edu.tw; 7 Department of Physical Education, Fu Jen Catholic University, New Taipei City 242, Taiwan; 8 Graduate Institute of Sport Coaching Science, Chinese Culture University, Taipei City 111, Taiwan; sfckl54872@yahoo.com.tw; 9 Artificial Intelligence Development Center, Fu Jen Catholic University, New Taipei City 242, Taiwan; 10 Innovation Lab, H2U Corporation, New Taipei City 231, Taiwan; changchien.ws@gmail.com; 11 Research and Development Center for Physical Education, Health, and Information Technology, Fu Jen Catholic University, New Taipei City 242, Taiwan; 12 Office of Physical Education, Fu Jen Catholic University, New Taipei City 242, Taiwan

**Keywords:** physical fitness, body fat, adiposity, adults

## Abstract

In terms of public health, obesity and overweight have become major concerns worldwide. Nevertheless, regarding body composition, it is important to have a more precise understanding of the fat-to-muscle ratio. Hence, this study aimed to adopt bioelectrical impedance measurements to test body fat percentage (BF%) and to determine the associations between health-related physical fitness and both body fat (BF) distribution and BF obesity risk in Taiwanese adults. We conducted a cross-sectional study and reviewed data derived from Taiwan’s Scientific Physical Fitness Survey. From the database, responses from 17,970 participants aged 23–64 years were collected in this study. Each participant completed a series of health-related physical fitness measurements, including cardiorespiratory fitness (3 min of a progressive knee-up and step (3MPKS) test), muscular fitness (hand-grip strength), and flexibility (sit-and-reach test). The BF% of each participant was assessed using the bioelectrical impedance analysis method. BF% was negatively associated with low performance on the 3MPKS *(β* = 11.314, *p* < 0.0001 for men; *β* = 12.308, *p* < 0.0001 for women), hand-grip strength (*β* = 2.071, *p* < 0.0001 for men; *β* = 0.859, *p* < 0.0001 for women), and sit-and-reach (*β* = 0.337, *p* = 0.008 for women) tests but was positively associated with sit-and-reach (*β* = −0.394, *p* = 0.004 for men). A risk of BF obesity for low performance of 3MPKS (odds ratio (OR) = 26.554, *p* < 0.0001 for men; OR = 25.808, *p* < 0.0001 for women), hand-grip strength (OR = 1.682, *p* < 0.0001 for men; OR = 1.234, *p* < 0.0001 for women), and sit-and-reach (OR = 1.142, *p* = 0.007 for women) tests was observed. These results suggest that low levels of cardiorespiratory fitness, muscular fitness, and flexibility are associated with an increased risk of BF obesity.

## 1. Introduction

Worldwide, obesity and overweight have increased exponentially since 1975; in 2016, more than 1.9 billion adults (39%) were found to be overweight and over 650 million (13%) adults were found to be obese [1]. In Taiwan, according to the 2013–2016 “National Nutrition and Health Status Change Survey”, the prevalence of being overweight or obese among adults was 45.4% (53.4% for men and 38.3% for women), and the prevalence had increased by nearly 10% compared to a decade ago [2]. Being overweight or obese is defined as having excess adiposity that impairs health [1]. Moreover, the increased risks of metabolic and cardiovascular diseases are associated with an increase in obesity [3], thus leading to huge medical costs and a decline in quality of life [3,4].

Generally, body mass index (BMI) is used to easily define whether an individual is obese or not; however, it cannot distinguish the proportions of fat and muscle for individuals [5,6]. For instance, despite a normal BMI, it is still possible to have excess body fat, which is known as “normal-weight obesity syndrome” [7]. A past study indicated that normal-weight obese adults are insulin-resistant, hyperinsulinemic, and have type 2 diabetes mellitus, as well as premature coronary heart disease [8]. Furthermore, compared to normal-weight lean individuals, normal-weight obese individuals have a higher risk of developing metabolic syndrome, cardiometabolic dysfunction, and death [7]. Moreover, cardiovascular disease and metabolic syndrome are thought to be related to health-related physical fitness such as cardiorespiratory and muscular fitness [9,10,11,12]. As such, numerous studies have shown that a high BMI and/or obesity are associated with poor physical fitness due to higher body fat and lower skeletal muscle mass, using bioelectrical impedance and dual X-ray absorptiometry (DEXA) [13,14,15].

In terms of public health, obesity and being overweight have become major concerns worldwide. Nevertheless, regarding body composition, it is important to have a more precise understanding of the fat-to-muscle ratio. Hence, this study aimed to adopt bioelectrical impedance measurements to test body fat percentage (BF%) and to determine the associations between health-related physical fitness and both body fat (BF) distribution and BF obesity risk in Taiwanese adults.

## 2. Materials and Methods

### 2.1. Participants and Study Design

The cross-sectional data of this study were obtained from the 2017 Taiwan Scientific Physical Fitness Survey (TSPFS), which is an annual national survey conducted to examine the changes in the health-related physical fitness status of Taiwanese individuals. This survey was conducted by the Sports Administration, Ministry of Education in Taiwan. A convenience sampling scheme was employed, and the participants were recruited from 18 scientific physical fitness test stations. This survey included face-to-face interviews followed by a standardized structural questionnaire, anthropometric measurements, and health-related physical fitness tests that were conducted by trained examiners and medical specialists (typically nurses or doctors). The health-related physical fitness tests were conducted after pretest health screenings (screenings for health limitations affecting eligibility for participation in health-related physical fitness tests. The participants then completed a modified Physical Activity Readiness Questionnaire [16]. In this study, we included participants who fulfilled the following criteria: (1) 23–64 years of age and (2) Taiwanese. The exclusion criteria were as follows: (1) participants with systolic blood pressure ≥140 mmHg and/or diastolic blood pressure ≥90 mmHg, and (2) participants with heart disease, hypertension, chest pain, vertigo, and/or musculoskeletal disorders. Finally, 17,970 adult participants (7907 men and 10,063 women) aged 23–64 years were included in the present study (96.14% response rate). This study was conducted in accordance with the Declaration of Helsinki, and the protocol was approved by the Institutional Review Board of Fu Jen Catholic University in Taiwan (FJU-IRB C110113).

### 2.2. Data Collection

The participants’ sociodemographic characteristics, such as age, gender, education level, occupation condition, monthly income, marital status, and relationship status, were obtained via face-to-face interviews using a structured questionnaire published in previous studies [17,18]. The participants were divided into education levels of elementary school or lower, junior or senior school, and college or higher; in terms of occupation status, the participants were divided into currently employed, nonemployed, and other groups. Monthly income was categorized as ≤20,000 New Taiwan dollar (NTD), NTD 20,001–40,000, and ≥NTD 40,001. Marital status was categorized as never married, married, and divorced/separated/widowed. Furthermore, relationship status was categorized as living with someone and not living with someone.

The participants’ anthropometric measurements were performed before the health-related physical fitness tests. The anthropometric indices included body height and weight. During the anthropometric measurements, all of the participants had their shoes and heavy clothes removed and were instructed to stand erect with their abdomen relaxed, arms at their sides, and feet together. Body weight (in kilograms) and height (in meters) were measured to the nearest 0.1 kg and 0.1 cm, respectively, with an electronic height–weight scale. BMI was calculated as body mass in kilograms divided by the square of height in meters (kg/m^2^).

### 2.3. BF% Measurements and Definition of BF Obesity

Following the anthropometric measurements, the BF% of each participant was assessed via a bioelectrical impedance analysis (BIA) using three devices [19,20,21]: (1) an Omron handheld impedance device (Omron model HBF-306, Hoffman Estates, IL, USA) for bipolar arm–arm impedance, (2) a Tanita device (Tanita model TBF300A, Arlington Heights, IL, USA) for bipolar leg–leg impedance, and (3) an InBody 770 (InBody Model 770, Cerritos, CA, USA). These anthropometric index and body fat measurements were conducted by well-trained examiners who attended a regional training seminar and who passed a certification test on standardized procedures, as reported in previous studies [17,18,22]. Measurements with all of the devices were performed in accordance with the manufacturers’ instructions. BF obesity was classified according to the cut-off BF% point classification by gender for Taiwanese adults, which was adopted as suggested by the Health Promotion Administration, Ministry of Health and Welfare in Taiwan. The BF% value was calculated as ≥25% for men and ≥30% for women, which is considered the classification of BF obesity [5,23].

### 2.4. Health-Related Physical Fitness Measurements

To determine the health-related physical fitness status of adults, we assessed three main components of health-related physical fitness based on three measurements: cardiorespiratory fitness (3 min progressive knee-up and step (3MPKS) test), muscular fitness (hand-grip-strength test), and flexibility (sit-and-reach test). The measurements and the abovementioned data were recorded by well-trained examiners who had attended an official training seminar and who had passed a certification test on standardized procedures. The content and process of the health-related physical fitness measurements were explained to the participants, and they had 10 min to warm up so that their optimum performance could be achieved. All of the participants were assessed in the following order with sufficient rest (3–5 min) between measurements: Hand-grip-strength, sit-and-reach, and 3MPKS tests. The details of the 3MPKS, hand-grip-strength, and sit-and-reach tests were strictly performed according to the Scientific Physical Fitness Test manual, as described in the following statement.

#### 2.4.1. MPKS Test

Cardiorespiratory fitness was measured as VO_2_max (mL·kg^−1^·min^−1^) using the 3MPKS test [24,25]. Before performing the 3MPKS test, the participants were required to wear a heart-rate monitor with a chest strap to monitor their HR during exercise testing. While wearing the HR monitor, the participants stood while the midway point between their patella and iliac crest was measured as the target height for lifting the knees and was marked by colored tape. Once the test had been initiated, the participants were asked to match a rhythm produced by an electronic metronome while stepping in place and raising the knee to the marked height with each step. The 3MPKS test started with 96 steps per minute (SPM), and the rate was increased by 24 SPM every minute. If the participants were unable to maintain the rhythm, they could run instead of walk for up to 3 min. If the participants were unable to lift the knees to the required height or if they could not follow the rhythm for 30 s, then the test session was terminated and the results were eliminated from the analysis. For safety concerns, the participants had to maintain the step rate at 80 SPM for a cool-down period of 30 s before resting in a standing position. The recorded data contained the HR at the beginning of exercise testing (HR0); at the first (HR1), second (HR2), and third minutes (HR3) during the exercise testing; and at the first minute postexercise testing (HR4).

The predicted VO_2_max was calculated as follows [24]: 72.334 − 0.261 × age + 4.366 × gender − 0.448 × fat% − 0.134 × HR0 − 0.082 × (∆HR3 − HR0) + 0.073 × (∆HR3 − HR4).

#### 2.4.2. Hand-Grip Strength

Muscular fitness was measured to the nearest 0.1 kg as the maximal isometric hand-grip strength (in kilograms) using a digital hand dynamometer (T.K.K.5401 Grip-D, Takei Scientific Instruments Co., Ltd., Niigata City, Japan). The participants were asked to stay in a standing position and to keep one arm straight and parallel to the body [26]. They were requested to hold the dynamometer with their dominant hand and to squeeze the handle as hard as possible for 3 s. The test was performed twice, and the average value was used for data analysis.

#### 2.4.3. Sit-and-Reach Test

Flexibility was measured as low back and hamstring flexibility (centimeters) using a sit-and-reach test [27]. From a seated position with their feet placed flat against a prepared box with a zero point at −25 cm with respect to the feet, the participants had to gradually push a ruler on the box while trying to reach as far forward as possible and while always keeping their knees straight. Bouncing movements were not allowed. The test was performed twice, and the best result was used for data analysis.

### 2.5. Statistical Analyses

The data were analyzed via SAS statistical software (version 9.4; SAS Institute, Inc., Cary, NC, USA). Differences in demographic characteristics, anthropometric variables, and HRPF measurements between the with/without BF obesity group were analyzed using Student’s *t*-tests or chi-square tests. A multiple linear regression analysis with BF% as the dependent variable was used to examine the associations between HRPF measurements and BF% after adjustments for potential confounders, such as age, education, occupation, monthly income, marital status, and relationship status. To examine the risk relationship between HRPF measurements and both BF% and BF obesity status, two different categories (50th percentiles) were applied for each HRPF measurement according to gender. The low quartile was composed of participants who had the best performance in each HRPF measurement, and it was assigned as the reference group for further analysis. Unconditional logistic regression analyses were conducted to evaluate the linear associations between cardiorespiratory fitness, muscular fitness, and flexibility and BF obesity risks. All of the regression models were adjusted for age, education, occupation, monthly income, marital status, relationship status, and other HRPF measurements. Afterward, the adjusted odds ratios (ORs) with 95% confidence intervals (CIs) were calculated. All of the data are expressed as the means ± standard deviation or frequency (percentage). The significance level adopted to reject the null hypothesis was *p* < 0.05.

## 3. Results

In total, 7907 men and 10,063 women were included in this study. Table 1 lists the demographic characteristics of the study participants, as classified by their BF obesity status. A total of 3389 men and 5136 women had BF obesity, whereas 4518 men and 4927 women had non-BF obesity. Both sexes in the BF obesity group were older and had higher BMI, WC, HC, and WHR values. Significant differences were found in education, current employment status, income level status, marital status, and relationship status among both the men and women.

Table 2 presents a comparison of BF obesity status differences by various health-related physical fitness measurements among adults in Taiwan. The results indicate that the non-BF obesity group had significantly higher results for all of the physical fitness tests without the sit-and-reach test in men.

Table 3 presents the results of the regression coefficients for predicting BF% using different health-related physical fitness measurements. In men, adjusted for age, education, occupation, monthly income, marital status, and other physical fitness measurements, the power was decreased in the 3MPKS (*β* = −1.565) and sit-and-reach tests (β = 0.027) and increased in the grip-strength test (*β* = −0.104). However, in women, after adjusting for age, education, occupation, monthly income, marital status, and other physical fitness measurements, the power was decreased in the 3MPKS (*β* = −1.538) and grip-strength (*β* = −0.066) tests.

Table 4 presents the results of the regression coefficients for predicting the BF% of the health-related physical fitness measurements. In men, after adjusting for age, education, occupation, monthly income, marital status, and other physical fitness measurements, the power significantly increased with the 3MPKS (*β* = 11.314) and sit-and-reach tests (*β* = −0.394) and decreased with the grip-strength test (*β* = 2.071). However, in women, after adjusting for age, education, occupation, monthly income, marital status, and other health-related physical fitness measurements, the power significantly increased with the 3MPKS (*β* = 12.038) and grip-strength tests (*β* = 0.859) and decreased in the sit-and-reach test (*β* = 0.337).

Table 5 presents the multivariate-adjusted ORs for BF obesity in relation to the 50th percentiles of the health-related physical fitness measurements after adjustments for potential confounders. For the 3MPKS test results, Model 2 shows that the male participants with results below the baseline 41.65 had a higher risk of BF obesity than those with test results ≥41.65 (OR = 26.554). The female participants with results below the baseline 34.42 also had a higher risk of BF obesity than those with test results ≥34.42 (OR = 25.808). For the grip-strength-test results, Model 2 shows that the male participants with results below the baseline 42.00 had a higher risk of BF obesity than those with a test result ≥42.00 (OR = 1.682). The female participants with results below the baseline 25.40 also had a higher risk of BF obesity than those with a test result ≥25.40 (OR = 1.234). For the sit-and-reach test results, Model 2 shows that there was no significant difference in BF obesity between the participants with results below the baseline 29.00 and those with test results ≥29.00 in males (OR = 0.911). However, females with a result below the baseline 27.00 had a higher risk of BF obesity than those with a test result ≥27.00 (OR = 1.142).

## 4. Discussion

In this study, the aim was to determine the relationship between health-related physical fitness and BF obesity risk using bioelectrical impedance in Taiwanese adults. This study adopted a large-scale national cross-sectional design and was conducted with a national database. The results of this study showed a significant relationship between health-related physical fitness and BF obesity risk in all of the fitness measurements. Furthermore, in the worst fitness interval, it was most strongly associated with BF obesity risk in both sexes.

We found that adults had lower cardiorespiratory fitness, which has a strong correlation with being BF obese. Most previous studies have used BMI as the standard for obesity; a past study indicated that changes in cardiorespiratory fitness are affected as BMI increases [28]. The measurements of this study, with the use of a more precise instrument via bioelectrical impedance, are also consistent with previous studies. Generally, cardiopulmonary fitness is considered to be highly correlated with cardiovascular disease. Different studies [29,30,31,32] have documented an association between obesity and cardiovascular disease. This study is consistent with past studies, demonstrating that cardiovascular fitness, as tested via 3MPKS, can effectively predict the obesity risk, which is similar to previous studies.

The results of this study showed that muscle strength and endurance are measured via grip strength, which is an important indicator of body fat and obesity. It is important for obese individuals who are recommended to perform resistance training two to three times a week [33,34,35] to maintain or increase muscle strength and to decrease body mass [34,36]. A past study indicated that after 16 weeks of a resistant exercise intervention, elderly women with obesity had improved adiposity indices and increased muscle strength and functional ability [37]. Furthermore, muscular flexibility was confirmed to predict body fat obesity in both male and female participants. However, no previous studies have been conducted on the relationship between muscular strength and flexibility and body fat obesity; thus, future research can determine whether muscle flexibility or strength can be solely used as a main indicator of obesity.

The strength of the present study lies in using a large sample size and representative data for analysis, providing an idea of how cross-sectional changes occur in health-related physical fitness and obesity risk in Taiwanese adults. However, there are still some limitations that need to be discussed. First, due to the fact that this study used a cross-sectional design, the lack of longitudinal data made it too difficult to infer causality relationships. Second, our questionnaire did not include chronic diseases such as cardiovascular disease, hypertension, diabetes, and metabolic syndrome, which may have interfered with health-related physical fitness performance. Third, this study population included Taiwanese adults between the ages of 23 and 64 years, meaning the results cannot be generalized to other age groups or a variety of different demographic characteristics. Additionally, differences in adult ages were unable to be assessed in the present design. Hence, we recommend that future studies adopt a longitudinal study design and recruit more different participants, or a specific age population, to understand this issue more comprehensively.

## 5. Conclusions

In summary, the purpose of the present study was to determine the relationship between health-related physical fitness and BF obesity risk in Taiwanese adults. The results suggested that low levels of cardiorespiratory fitness, muscular fitness, and flexibility are associated with an increased risk of BF obesity. However, the mechanism by which physical fitness affects BF obesity is still unclear, and future studies can further investigate this issue.

## Figures and Tables

**Table 1:** Anthropometric and sociodemographic characteristics of the study participants, who were Taiwanese adults, with or without body fat obesity. ^a^

Variables	Men (*n* = 7907)			Women (*n* = 10,063)		
	BF Obesity (*n* = 3389)	Non-BF Obesity (*n* = 4518)	*p-*Value	BF Obesity (*n* = 5136)	Non-BF Obesity (*n* = 4927)	*p-*Value
Age (years)	38.45 ± 11.32	36.59 ± 10.87	<0.0001 *	37.87 ± 11.81	37.09 ± 10.34	<0.0001 *
Height (cm)	170.24 ± 6.64	172.70 ± 6.03	<0.0001 *	158.99 ± 5.78	160.62 ± 5.73	<0.0001 *
Body weight (kg)	75.42 ± 12.41	69.89 ± 9.25	<0.0001 *	59.42 ± 9.20	54.17 ± 7.90	<0.0001 *
BMI (kg/m^2^)	25.95 ± 3.53	23.41 ± 2.61	<0.0001 *	23.49 ± 3.28	20.96 ± 2.53	<0.0001 *
WC (cm)	84.00 ± 9.27	80.62 ± 7.95	<0.0001 *	77.28 ± 8.81	72.67 ± 7.61	<0.0001 *
HC (cm)	98.30 ± 7.20	96.33 ± 8.08	<0.0001 *	95.10 ± 8.08	90.97 ± 7.73	<0.0001 *
WHR	0.85 ± 0.06	0.84 ± 0.04	<0.0001 *	0.81 ± 0.06	0.80 ± 0.05	<0.0001 *
FM (kg)	24.06 ± 4.07	13.35 ± 3.90	<0.0001 *	22.28 ± 4.20	13.35 ± 3.13	<0.0001 *
FFM (kg)	51.35 ± 10.92	56.54 ± 6.90	<0.0001 *	37.14 ± 7.71	40.81 ± 6.00	<0.0001 *
%BF	32.41 ± 6.02	18.89 ± 4.16	<0.0001 *	37.85 ± 6.96	24.55 ± 3.97	<0.0001 *
Education (%)			<0.0001 *			<0.0001 *
Elementary school or lower	1.1	0.1		2.1	0.7	
Junior or senior high school	14.9	4.8		16.2	7.6	
College or higher	84.0	95.0		81.6	91.7	
Currently employed (%)			<0.0001 *			<0.0001 *
Yes	87.2	91.7		81.8	88.7	
No	10.8	4.4		16.3	7.9	
Other	2.0	3.9		1.8	3.5	
Income level (%)			<0.0001 *			<0.0001 *
≤NTD 20,000	11.1	6.1		15.5	8.7	
NTD 20,001–40,000	29.0	21.5		37.1	36.2	
≥NTD 40,001	59.9	72.4		47.4	55.1	
Marital status (%)			<0.0001 *			<0.0001 *
Never married	34.6	47.8		34.8	46.4	
Married	59.1	46.9		59.4	47.0	
Divorced/separated/widowed	6.3	5.3		5.8	6.5	
Relationship status (%)			<0.0001 *			<0.0001 *
Living with someone	84.0	78.2		87.8	83.5	
Not living with someone	16.0	21.8		12.2	16.5	

BF, body fat; BMI, body mass index; HC, hip circumference; FFM, free fat mass; FM, fat mass; NTD, New Taiwan dollar; SD, standard deviation; WC, waist circumference; WHR, waist-to-hip ratio. BF obesity, %BF ≥ 25% in men and ≥30% in women; non-BF obesity, %BF <25% in men and %BF <30% in women. ^a^ Values expressed as the mean ± SD or percentage (%). * Significantly different BF and non-BF obesity groups by Student’s *t*-test or chi-square test at *p* < 0.05.

**Table 2:** Scientific physical fitness measurements on body fat obesity in Taiwanese adults.

Variables	Men (*n* = 7907)			Women (*n* = 10,063)		
	BF Obesity (*n* = 3389)	Non-BF Obesity (*n* = 4518)	*p-*Value	BF Obesity (*n* = 5136)	Non-BF Obesity (*n* = 4927)	*p-*Value
3MPKS (mL/kg/min)	38.33 ± 3.85	44.56 ± 4.39	<0.0001 *	31.83 ± 4.11	37.66 ± 4.39	<0.0001 *
Grip strength (kg)	38.70 ± 9.50	43.60 ± 7.86	<0.0001 *	25.01 ± 5.88	26.05 ± 5.78	<0.0001 *
Sit-and-reach test (cm)	21.73 ± 10.00	21.28 ± 9.54	0.041 *	26.84 ± 9.97	27.76 ± 10.86	<0.0001 *

3MPKS, 3 min progressive knee-up and step; BF, body fat; SD, standard deviation. BF obesity, %BF ≥25% in men and ≥30% in women; non-BF obesity, %BF <25% in men and %BF <30% in women. Values are expressed as the mean ± SD. * Significantly different BF and non-BF obesity groups by Student’s *t*-test at *p* < 0.05.

**Table 3:** Multiple linear regression coefficients for predicting body fat percentage using different physical fitness measurements.

Variables	Model 1 (Unadjusted)			Model 2 (Adjusted) ^a^		
	*β*	SE	*p-*Value	*β*	SE	*p-*Value
**Men (*n* = 7907)**						
3MPKS (mL/kg/min)	−1.085	0.013	<0.0001 *	−1.565	0.012	<0.0001 *
Grip strength (kg)	−0.187	0.007	<0.0001 *	−0.104	0.006	<0.0001 *
Sit-and-reach test (cm)	0.059	0.006	<0.0001 *	0.027	0.005	<0.0001 *
**Women (*n* = 10,063)**						
3MPKS (mL/kg/min)	−1.116	0.013	<0.0001 *	−1.538	0.012	<0.0001 *
Grip strength (kg)	−0.026	0.011	0.022 *	−0.066	0.009	<0.0001 *
Sit-and-reach test (cm)	−0.015	0.006	0.016 *	−0.007	0.005	0.166

3MPKS, 3 min progressive knee-up and step; SE, standard error; β, regression coefficient. ^a^ Adjusted for age, education, occupation, monthly income, marital status, relationship status, and other physical fitness measurements. * *p* < 0.05.

**Table 4:** Multiple linear regression coefficients for predicting body fat percentage using the 50th percentiles of physical fitness measurements.

Variables	Model 1 (Unadjusted)			Model 2 (Adjusted) ^a^		
	*β*	SE	*p-*Value	*β*	SE	*p-*Value
**Men**						
3MPKS (mL/kg/min)						
<41.65/ref.: ≥41.65	9.443	0.152	<0.0001 *	11.314	0.161	<0.0001 *
Grip strength (kg)						
<42.00/ref.: ≥42.00	2.545	0.152	<0.0001 *	2.071	0.140	<0.0001 *
Sit-and-reach test (cm)						
<21.00/ref.: ≥21.00	–0.773	0.150	<0.0001 *	–0.394	0.137	0.004 *
**Women**						
3MPKS (mL/kg/min)						
<34.42/ref.: ≥34.42	9.823	0.145	<0.0001 *	12.038	0.142	<0.0001 *
Grip strength (kg)						
<25.40/ref.: ≥25.40	0.562	0.145	<0.0001 *	0.859	0.128	<0.0001 *
Sit-and-reach test (cm)						
<27.00/ref.: ≥27.00	0.422	0.144	0.003 *	0.337	0.127	0.008 *

3MPKS, 3 min progressive knee-up and step; SE, standard error; β, regression coefficient. ^a^ Adjusted for age, education, occupation, monthly income, marital status, relationship status, and other physical fitness measurements. * *p* < 0.05.

**Table 5:** Multivariate logistic regression model for BF obesity in relation to the 50th percentiles of physical fitness measurements after adjustment for potential confounders.

Variables	Model 1 (Unadjusted)			Model 2 (Adjusted) ^a^		
	OR	95% CI	*p-*Value	OR	95% CI	*p-*Value
**Men (*n* = 7907)**						
3MPKS (mL/kg/min)						
<41.65/ref.: ≥41.65	10.511	9.435–11.711	<0.0001 *	26.554	22.718–31.038	<0.0001 *
Grip strength (kg)						
<42.00/ref.: ≥42.00	1.743	1.568–1.937	<0.0001 *	1.682	1.503–1.883	<0.0001 *
Sit-and-reach test (cm)						
<21.00/ref.: ≥21.00	0.845	0.760–0.939	0.0002 *	0.911	0.815–1.019	0.104
**Women (*n* = 10,063)**						
3MPKS (mL/kg/min)						
<34.42/ref.: ≥34.42	10.316	9.406–11.315	<0.0001 *	25.808	22.544–29.545	<0.0001 *
Grip strength (kg)						
<25.40/ref.: ≥25.40	1.124	1.024–1.232	0.014 *	1.234	1.119–1.360	<0.0001 *
Sit-and-reach test (cm)						
<27.00/ref.: ≥27.00	1.148	1.047–1.259	0.003 *	1.142	1.037–1.258	0.007 *

3MPKS, 3 min progressive knee-up and step; CI, confidence interval; OR, odds ratio. BF obesity, %BF ≥ 25% in men and ≥30% in women; non-BF obesity, %BF < 25% in men and %BF < 30% in women. ^a^ Adjusted for age, education, occupation, monthly income, marital status, relationship status, and other physical fitness measurements. * *p* < 0.05.

## Data Availability

The datasets generated and analyzed in this study are not publicly available, but are available from the corresponding authors upon reasonable request.

## References

[1] World Health Organization (2016). Obesity and Overweight Fact Sheet.

[2] Health Promotion Administration, Ministry of Health and Welfare Health Promotion Statistical Report. https://www.hpa.gov.tw/Pages/List.aspx?nodeid=268.

[3] Lavie C.J., Laddu D., Arena R., Ortega F.B., Alpert M.A., Kushner R.F. (2018). Healthy weight and obesity prevention: JACC health promotion series. J. Am. Coll. Cardiol..

[4] Cawley J., Meyerhoefer C. (2012). The medical care costs of obesity: An instrumental variables approach. J. Health Econ..

[5] Okorodudu D.O., Jumean M.F., Montori V.M., Romero-Corral A., Somers V.K., Erwin P.J., Lopez-Jimenez F. (2010). Diagnostic performance of body mass index to identify obesity as defined by body adiposity: A systematic review and meta-analysis. Int. J. Obes..

[6] Gómez-Ambrosi J., Silva C., Galofré J.C., Escalada J., Santos S., Millán D., Valentí V., Rotellar F. (2012). Body mass index classification misses subjects with increased cardiometabolic risk factors related to elevated adiposity. Int. J. Obes..

[7] Oliveros E., Somers V.K., Sochor O., Goel K., Lopez-Jimenez F. (2014). The concept of normal weight obesity. Prog. Cardiovasc. Dis..

[8] Franco L.P., Morais C.C., Cominetti C. (2016). Normal-weight obesity syndrome: Diagnosis, prevalence, and clinical implications. Nutr. Rev..

[9] Mintjens S., Menting M.D., Daams J.G., van Poppel M.N., Roseboom T.J., Gemke R.J. (2018). Cardiorespiratory fitness in childhood and adolescence affects future cardiovascular risk factors: A systematic review of longitudinal studies. Sports Med..

[10] García-Hermoso A., Ramírez-Campillo R., Izquierdo M. (2019). Is muscular fitness associated with future health benefits in children and adolescents? A systematic review and meta-analysis of longitudinal studies. Sports Med..

[11] Adamu B., Sani M.U., Abdu A. (2006). Physical exercise and health: A review. Niger. J. Med..

[12] Ekelund U., Steene-Johannessen J., Brown W.J., Fagerland M.W., Owen N., Powell K.E., Bauman A., Lee I.M., Series L.P.A., Lancet Sedentary Behaviour Working Group (2016). Does physical activity attenuate, or even eliminate, the detrimental association of sitting time with mortality? A harmonised meta-analysis of data from more than 1 million men and women. Lancet.

[13] Bonney E., Ferguson G., Smits-Engelsman B. (2018). Relationship between body mass index, cardiorespiratory and musculoskeletal fitness among South African adolescent girls. Int. J. Environ. Res. Public Health.

[14] Lopes V.P., Malina R.M., Gomez-Campos R., Cossio-Bolaños M., Arruda M.D., Hobold E. (2019). Body mass index and physical fitness in Brazilian adolescents. J. Pediatr..

[15] Nichols S., O’Doherty A.F., Taylor C., Clark A.L., Carroll S., Ingle L. (2019). Low skeletal muscle mass is associated with low aerobic capacity and increased mortality risk in patients with coronary heart disease—A CARE CR study. Clin. Physiol. Funct. Imaging.

[16] Suni J.H., Miilunpalo S.I., Asikainen T.M., Laukkanen R.T., Oja P., Pasanen M.E., Bös K., Vuori I.M. (1998). Safety and feasibility of a health-related fitness test battery for adults. Phys. Ther..

[17] Lee P.F., Ho C.C., Yeh D.P., Hung C.T., Chang Y.C., Liu C.C., Tseng C.Y., Hsieh X.Y. (2020). Cross-sectional associations of physical fitness performance level and sleep duration among older adults: Results from the national physical fitness survey in Taiwan. Int. J. Environ. Res. Public Health.

[18] Liao Y., Tsai H.H., Wang H.S., Ling C.P., Wu M.C., Chen J.F. (2016). Traveling by private motorized vehicle and physical fitness in Taiwanese adults. Int. J. Behav. Med..

[19] Anderson L.J., Erceg D.N., Schroeder E.T. (2012). Utility of multifrequency bioelectrical impedance compared with dual-energy x-ray absorptiometry for assessment of total and regional body composition varies between men and women. Nutr. Res..

[20] Vasold K.L., Parks A.C., Phelan D.M., Pontifex M.B., Pivarnik J.M. (2019). Reliability and validity of commercially available low-cost bioelectrical impedance analysis. Int. J. Sport Nutr. Exerc. Metab..

[21] Von Hurst P.R., Walsh D.C., Conlon C.A., Ingram M., Kruger R., Stonehouse W. (2016). Validity and reliability of bioelectrical impedance analysis to estimate body fat percentage against air displacement plethysmography and dual-energy X-ray absorptiometry. Nutr. Diet..

[22] Liu C.M., Lin K.F. (2007). Estimation of VO2max: A comparative analysis of post-exercise heart rate and physical fitness index from 3-minute step test. JESF.

[23] Kim J.Y., Han S.H., Yang B.M. (2013). Implication of high-body-fat percentage on cardiometabolic risk in middle-aged, healthy, normal-weight adults. Obesity.

[24] Chung Y.-C., Huang C.-Y., Wu H.-J., Kan N.-W., Ho C.-S., Huang C.-C., Chen H.-T. (2021). Predicting maximal oxygen uptake from a 3-minute progressive knee-ups and step test. PeerJ.

[25] Li F., Chang C.-H., Chung Y.-C., Wu H.-J., Kan N.-W., ChangChien W.-S., Ho C.-S., Huang C.-C. (2021). Development and validation of 3 min incremental step-in-place test for predicting maximal oxygen uptake in home settings: A submaximal exercise study to assess cardiorespiratory fitness. Int. J. Environ. Res. Public Health.

[26] Bohannon R.W., Magasi S.R., Bubela D.J., Wang Y.C., Gershon R.C. (2012). Grip and knee extension muscle strength reflect a common construct among adults. Muscle Nerve.

[27] Ayala F., de Baranda P.S., Croix M.D.S., Santonja F. (2012). Absolute reliability of five clinical tests for assessing hamstring flexibility in professional futsal players. J. Sci. Med. Sport.

[28] Ross R., Katzmarzyk P.T. (2003). Cardiorespiratory fitness is associated with diminished total and abdominal obesity independent of body mass index. Int. J. Obes..

[29] Apovian C.M., Gokce N. (2012). Obesity and cardiovascular disease. Circulation.

[30] Zalesin K.C., Franklin B.A., Miller W.M., Peterson E.D., McCullough P.A. (2008). Impact of obesity on cardiovascular disease. Endocrinol. Metab. Clin. N. Am..

[31] Manrique-Acevedo C., Chinnakotla B., Padilla J., Martinez-Lemus L.A., Gozal D. (2020). Obesity and cardiovascular disease in women. Int. J. Obes..

[32] Dwivedi A.K., Dubey P., Cistola D.P., Reddy S.Y. (2020). Association between obesity and cardiovascular outcomes: Updated evidence from meta-analysis studies. Curr. Cardiol. Rep..

[33] Mitchell S., Shaw D. (2015). The worldwide epidemic of female obesity. Best Pract. Res. Clin. Obstet. Gynaecol..

[34] Donnelly J.E., Blair S.N., Jakicic J.M., Manore M.M., Rankin J.W., Smith B.K. (2009). American College of Sports Medicine Position Stand. Appropriate physical activity intervention strategies for weight loss and prevention of weight regain for adults. Med. Sci. Sports Exerc..

[35] Shaw K.A., Gennat H.C., O’Rourke P., Del Mar C. (2006). Exercise for overweight or obesity. Cochrane Database Syst. Rev..

[36] Swift D.L., Johannsen N.M., Lavie C.J., Earnest C.P., Church T.S. (2014). The role of exercise and physical activity in weight loss and maintenance. Prog. Cardiovasc. Dis..

[37] De Oliveira Silva A., Dutra M.T., de Moraes W.M.A.M., Funghetto S.S., de Farias D.L., Dos Santos P.H.F., Vieira D.C.L., da Cunha Nascimento D., Orsano V.S.M., Schoenfeld B.J. (2018). Resistance training-induced gains in muscle strength, body composition, and functional capacity are attenuated in elderly women with sarcopenic obesity. Clin. Interv. Aging.

